# Targeted Delivery of Cell Penetrating Peptide Virus-like Nanoparticles to Skin Cancer Cells

**DOI:** 10.1038/s41598-018-26749-y

**Published:** 2018-05-31

**Authors:** Bee Koon Gan, Chean Yeah Yong, Kok Lian Ho, Abdul Rahman Omar, Noorjahan Banu Alitheen, Wen Siang Tan

**Affiliations:** 1 Institute of Bioscience, Universiti Putra Malaysia, 43400 UPM Serdang, Selangor Malaysia; 2 Department of Pathology, Faculty of Medicine and Health Sciences, Universiti Putra Malaysia, 43400 UPM Serdang, Selangor Malaysia; 3 Department of Veterinary Pathology & Microbiology, Faculty of Veterinary Medicine, Universiti Putra Malaysia, 43400 UPM Serdang, Selangor Malaysia; 4 Department of Cell and Molecular Biology, Faculty of Biotechnology and Biomolecular Sciences, Universiti Putra Malaysia, 43400 UPM Serdang, Selangor Malaysia; 5 Department of Microbiology, Faculty of Biotechnology and Biomolecular Sciences, Universiti Putra Malaysia, 43400 UPM Serdang, Selangor Malaysia

## Abstract

Skin cancer or cutaneous carcinoma, is a pre-eminent global public health problem with no signs of plateauing in its incidence. As the most common treatments for skin cancer, surgical resection inevitably damages a patient’s appearance, and chemotherapy has many side effects. Thus, the main aim of this study was to screen for a cell penetrating peptide (CPP) for the development of a targeting vector for skin cancer. In this study, we identified a CPP with the sequence NRPDSAQFWLHH from a phage displayed peptide library. This CPP targeted the human squamous carcinoma A431 cells through an interaction with the epidermal growth factor receptor (EGFr). Methyl-β-cyclodextrin (MβCD) and chlorpromazine hydrochloride (CPZ) inhibited the internalisation of the CPP into the A431 cells, suggesting the peptide entered the cells via clathrin-dependent endocytosis. The CPP displayed on hepatitis B virus-like nanoparticles (VLNPs) via the nanoglue successfully delivered the nanoparticles into A431 cells. The present study demonstrated that the novel CPP can serve as a ligand to target and deliver VLNPs into skin cancer cells.

## Introduction

Skin cancer or cutaneous carcinoma is a serious global public health problem that poses large economic burden to the society. A total of 1.6 million new cases of cutaneous malignancy with 12,190 deaths from skin cancer were reported by the American Cancer Society in 2012^1^. Squamous cell carcinoma (SCC) is one of the most common skin cancers which accounts for approximately 20% of non-melanocytic skin cancers^2^. SCC arises from epidermal keratinocytes and normally develops at skin areas which are frequently exposed to ultraviolet (UV), particularly on the face and arms. SCC can result in significant disfigurement and it can invade other tissues and cause death^3^. Surgical resection combined with chemotherapy represents the most common treatment for SCC. However, surgery would inevitably damage a patient’s appearance, and chemotherapy has many side effects due to non-specific distribution of chemotherapeutic drugs to normal cells. Therefore, there is an urgent need to develop a novel transdermal drug delivery system to minimize undesirable effects of therapeutic molecules to the normal cells while increase its permeation efficacy into the skin cancer cells.

Targeting therapy represents a potential treatment for SCC to overcome the drawbacks of current treatment strategies. Traditional targeting delivery mostly relies on monoclonal antibodies. Although specific, they are highly immunogenic and have low penetration rate into tumour cells^4^. Thus, peptide ligands which have low immunogenicity, high penetration rate and easy incorporation to delivery vehicles have become more favourable for specific delivery of therapeutic agents to tumours^4,5^. Cell penetrating peptides (CPPs) are peptides containing 5 to 30 residues, which interact specifically with cell surfaces and penetrate cell membranes without damaging the membranes^6^. CPPs have become increasingly popular for specific cell targeting delivery^7,8^. CPPs with high affinity and specificity towards their target receptors and cells can be identified from a phage displayed peptide library via biopanning^4,5,7,9–14^. In the present study, CPPs which internalised SCC were selected from a 12-residue phage displayed peptide library. Interestingly, the most dominant CPP with the sequence NRPDSAQFWLHH was found to target and internalise A431 cells but not normal skin cells. The receptor and entry mechanism of this CPP into A431 cells were studied.

This CPP could serve as a ligand to target and deliver virus-like nanoparticles (VLNPs) into skin cancer cells. To prove this hypothesis, truncated hepatitis B core antigen (tHBcAg) VLNPs were produced in *Escherichia coli*, purified, and used to display the CPP. These VLNPs have been employed to deliver green fluorescent protein (GFP), oligonucleotides and doxorubicin (DOX) into cancerous cells^7,15–17^. In the present study, this CPP was displayed on tHBcAg VLNPs via the nanoglue or a capsid binding peptide^7^. This nanoglue with the sequence SLLGRMKGA binds specifically and tightly at the spikes of tHBcAg VLNPs^18^. The CPP incorporated at the N-terminus of the nanoglue can be conjugated covalently and displayed easily on the VLNPs using N-hydroxysulfosuccinimide (sulfo-NHS) and 1-ethyl-3-(3-dimethylaminopropyl) carbodiimide hydrochloride (EDC). Fluorescence microscopy showed that the conjugated CPP successfully delivered the tHBcAg VLNPs into SCC A431 cells.

## Results

### Subtraction biopanning for the selection of A431 cell penetrating peptides (CPPs)

A431 CPPs were screened from a phage displayed dodecapeptide library through subtraction biopanning against normal human dermal fibroblast cell line (NHDF). A431 cells which had been infected with the subtracted phages from NHDF were then lysed to release the internalised phages. The number of internalised phages showed a gradual increase from 7.2 × 10^2^ pfu/mL to 1.68 × 10^4^ pfu/mL after 3 rounds of panning (Table 1). The inserts in the DNA molecules isolated from the internalised phages were sequenced and the deduced amino acid sequences are summarised in Table 2. The percentage of CPP with the sequence NRPDSAQFWLHH increased dramatically from 18.75% in round 1 to 95% in round 3 of biopanning.

**Table 1:** Enrichment of phages in three rounds of biopanning.

	1^st^ round	2^nd^ round	3^rd^ round
Phage Input (pfu/mL)	1.00 × 10^11^	1.00 × 10^11^	1.00 × 10^11^
Internalised Phage (pfu/mL)	7.20 × 10^2^	1.06 × 10^4^	1.68 × 10^4^
Yield (%)*	7.20 × 10^−7^	1.06 × 10^−5^	1.68 × 10^−5^
Enrichment Factor**	1.00	14.72	23.33

*Yield (%) = phage output/phage input × 100%.

**Enrichment factor was calculated as described in Zhang *et al*.^67^.

**Table 2:** Phages that internalised A431 cells and their deduced cell-penetrating peptides (CPPs) obtained from three rounds of biopanning.

	Peptide Sequence	Frequency	Percentage (%)
1^st^ Round	NRPDSAQFWLHH	3	18.75
	VDIHFLTGLGGG	1	6.25
	YAPLPIPLYPVA	1	6.25
	DEQVGSPPINRA	1	6.25
	DSSSMAEHSASF	1	6.25
	SGTHGDDRTTIT	1	6.25
	YKPSQLGSFGTT	1	6.25
	TGWTSHHSNLSR	1	6.25
	LMENRNPWVPDY	1	6.25
	ACSDECVRTRVP	1	6.25
	NLARGPVTDGSS	1	6.25
	FSTAHIVTPSFE	1	6.25
	YVGHHPFSDAWM	1	6.25
	EHDGLVVGTKKL	1	6.25
2^nd^ Round	NRPDSAQFWLHH	15	75
	SHWTLSPIAMTP	1	5
	GQSPHSYQPRTY	4	20
3^rd^ Round	NRPDSAQFWLHH	19	95
	SHITPLLNAAPF	1	5

**Figure 1:**
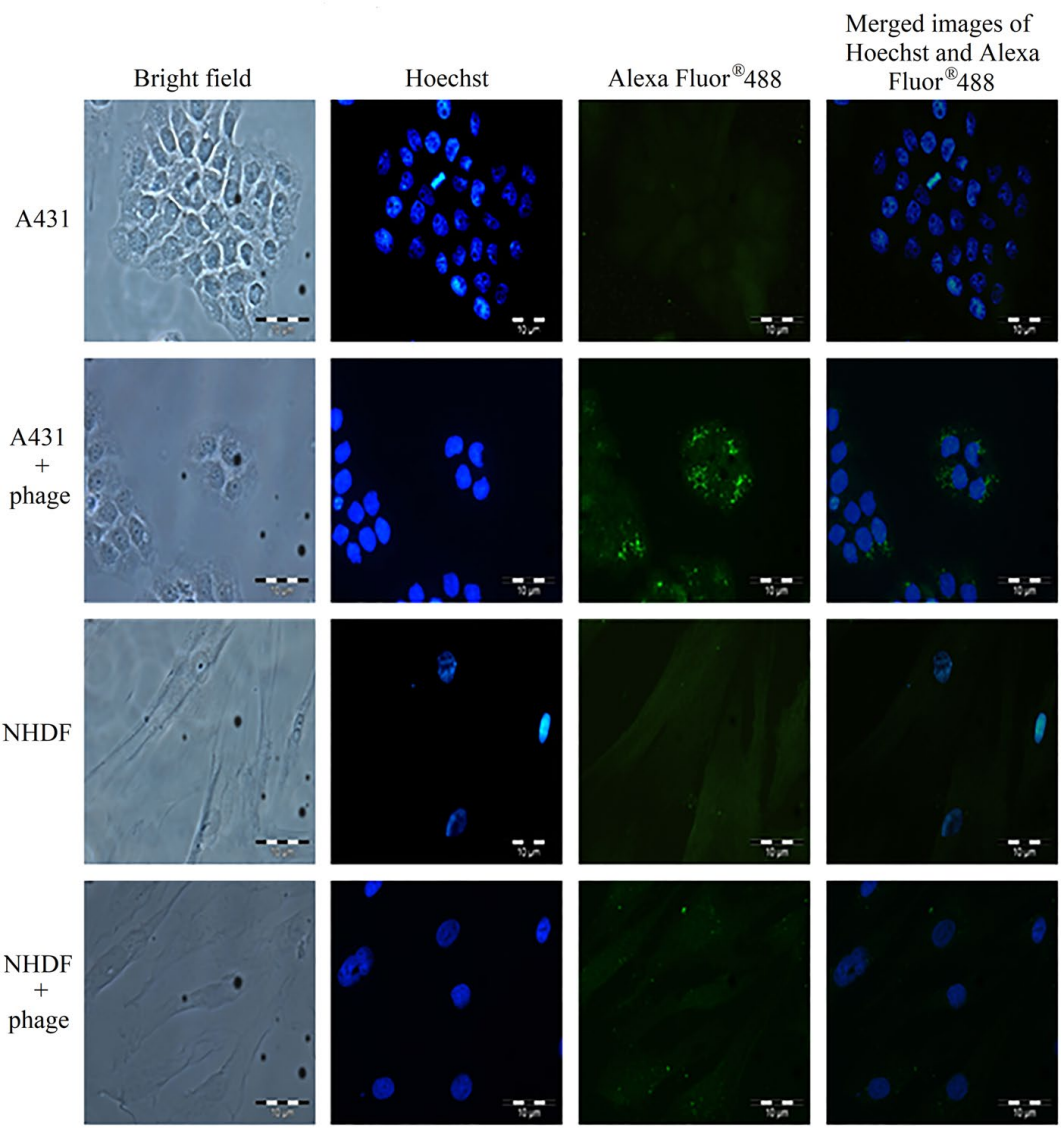
Analysis of the internalisation of phage clone into A431 and NHDF cells with immunofluorescence microscopy. The cell lines used are labelled on the left. A431 and NHDF cells without phage served as negative controls. Purified phage (1 × 10^12^ pfu) carrying the peptide NRPDSAQFWLHH was added to the cells. After incubation at 37 °C for 72 h, the phage was detected with the mouse anti-M13 monoclonal antibody, followed by the goat anti-mouse IgG conjugated to Alexa -Fluor^®^ 488.

The cell internalisation property of the isolated phage clone carrying the dominant peptide NRPDSAQFWLHH was verified by infecting A431 cells with the purified phage. The internalisation property was studied using immunofluorescence microscopy in which the internalised phage was detected by the mouse anti-M13 monoclonal antibody followed by the goat anti-mouse IgG conjugated to Alexa-Fluor^®^ 488. Figure 1 shows green fluorescent granules appeared in A431 cells after 72 h incubation, indicating the phage clone carrying peptide NRPDSAQFWLHH internalised the cells. For NHDF cells added with the phage, fluorescent signal was negligible as compared to that of A431 cells, demonstrating the isolated phage bearing the peptide sequence NRPDSAQFWLHH has a significantly higher rate of internalisation towards A431 cells compared with NHDF cells.

### Active energy-dependent uptake of phage NRPDSAQFWLHH by A431 cells

**Figure 2:**
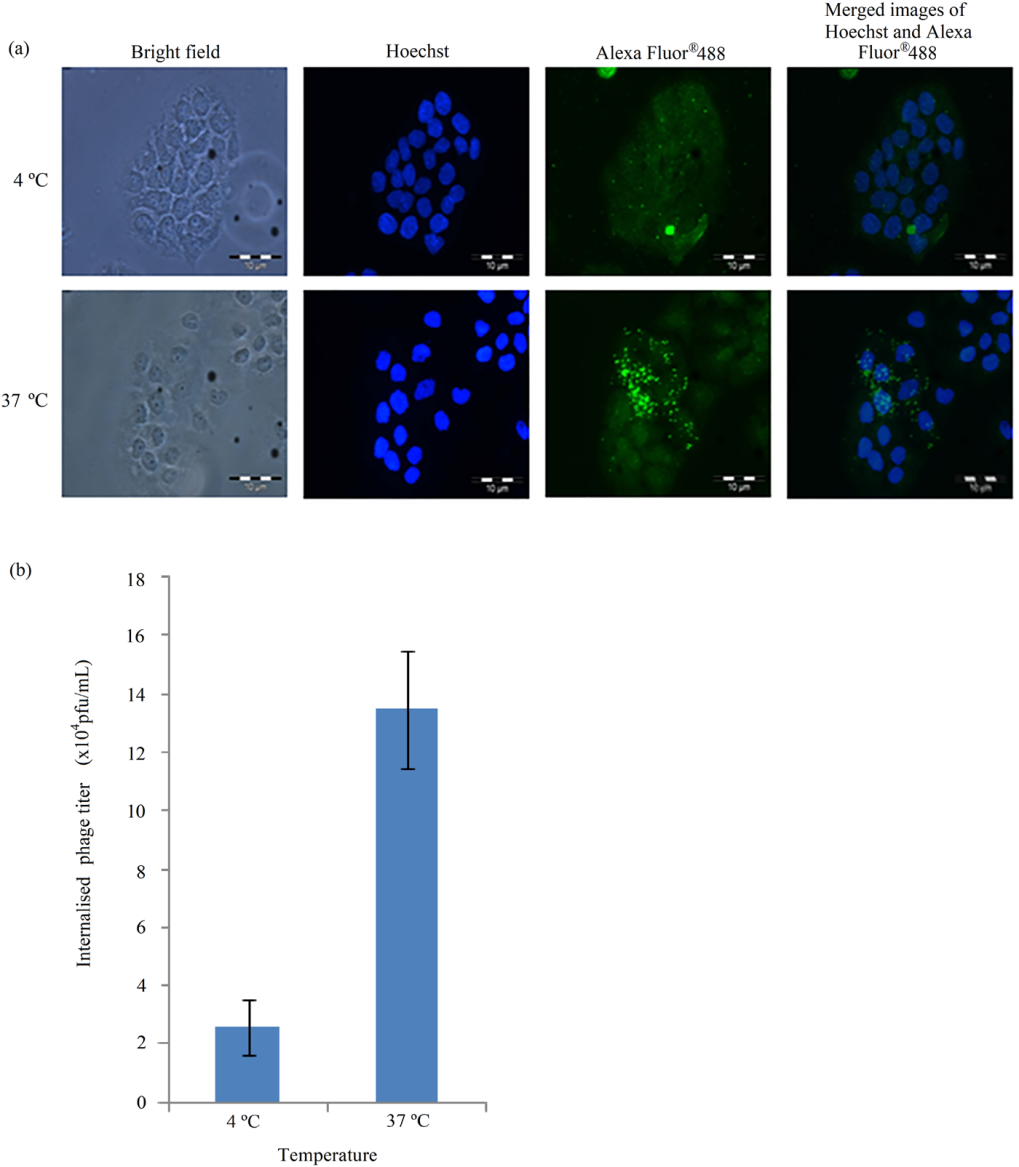
Energy dependent endocytosis of phage by A431 cells. (**a)** Immunofluorescence microscopic analysis. Purified phage (1 × 10^12^ pfu) carrying the peptide NRPDSAQFWLHH was added to A431 cells and incubated separately at 4 and 37 °C (labelled on the left of figure). After 12 h of incubation, the phage was detected with the mouse anti-M13 monoclonal antibody, followed by the goat anti-mouse IgG conjugated to Alexa-Fluor^®^ 488. (**b**) The amount of phage internalised A431 cells after 12 h of incubation at 4 °C and 37 °C was measured using the phage titration assay as described in the Materials and Methods. Data are expressed as mean ± standard deviation (n = 3).

In order to determine whether the internalisation of the isolated phage NRPDSAQFWLHH into A431 cells is an active and energy-dependent or passive process, the cells were incubated with the phage in parallel at 37 °C and 4 °C. Immunofluorescence microscopy showed that incubation of A431 cells with the phage NRPDSAQFWLHH at 4 °C resulted in a reduced fluorescent signal (Fig. 2a), and the titer of internalised phage as measured with the phage titration assay decreased to 19.15% (2.58 × 10^4^ pfu/mL) compared with that incubated at 37 °C (13.47 × 10^4^ pfu/mL) (Fig. 2b). This result indicates a strong inhibition of active transport which resulted in a drastic reduction in phage internalisation at 4 °C.

### Fusion phage and peptide NRPDSAQFWLHH competitive assay, and selective internalisation of the peptide into A431 cells

**Figure 3:**
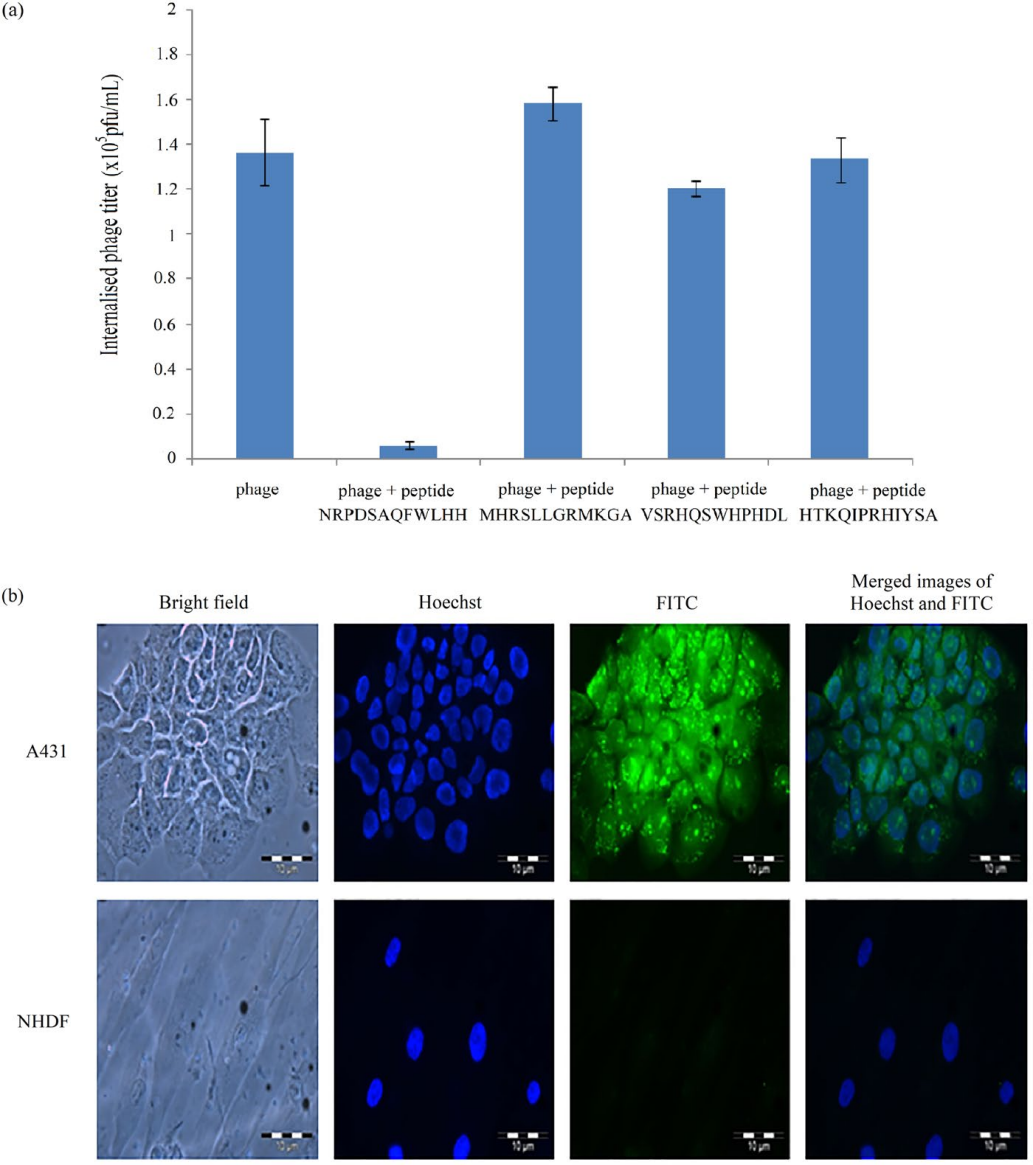
Synthetic peptide NRPDSAQFWLHH internalises A431 cells and competes with fusion phage. (**a**) Competition of phage and peptide NRPDSAQFWLHH to internalise A431 cells. Phage NRPDSAQFWLHH and peptide NRPDSAQFWLHH were added to the A431 cells and incubated for 12 h at 37 °C. The titer of the internalised phage was then determined. Peptide NRPDSAQFWLHH was able to inhibit phage NRPDSAQFWLHH from internalising A431 cells. However, the unrelated peptides (MHRSLLGRMKGA, VSRHQSWHPHDL and HTKQIPRHIYSA) which served as negative controls did not inhibit the phage from internalising the cells. Data are expressed as mean ± standard deviation (n = 3). (**b**) Synthetic peptide NRPDSAQFWLHH internalises A431 cells. A431 and NHDF cells were incubated with fluorescein-labelled peptide NRPDSAQFWLHH at 37 °C for 16 h. A significantly higher green fluorescent granules appeared in A431 cells as compared with NHDF cells.

To demonstrate the internalisation of phage NRPDSAQFWLHH into A431 cells is dependent on the fusion peptide NRPDSAQFWLHH instead of phage coat proteins, a competitive assay was carried out between the synthetic peptide NRPDSAQFWLHH with the fusion phage. Unrelated peptides MHRSLLGRMKGA, VSRHQSWHPHDL and HTKQIPRHIYSA were used as negative controls. As shown in Fig. 3a, co-incubation of A431 cells with the synthetic peptide and fusion phage resulted in a significant inhibition of the fusion phage from internalising the A431 cells by the peptide. The titer of internalised phage reduced by approximately 95.6%, from 1.367 × 10^5^ pfu/mL to 6 × 10^3^ pfu/mL, as compared with the incubation of A431 cells with the phage in the absence of peptide. Whereas, co-incubation of A431 cells with unrelated peptides, MHRSLLGRMKGA, VSRHQSWHPHDL and HTKQIPRHIYSA did not inhibit the internalisation of the fusion phage into A431 cells. The results demonstrate that peptide NRPDSAQFWLHH was indeed responsible for the internalisation of the phage into A431 cells.

To elucidate the selective internalisation activity of peptide NRPDSAQFWLHH into A431 cells, the peptide labelled with FITC was incubated in parallel with A431 and NHDF cells at 37 °C for 16 h. Internalisation of the peptide into these cells were examined using fluorescence microscopy. Figure 3b shows that in the absence of g3p protein and other phage coat proteins, free FITC-NRPDSAQFWLHH peptide was able to internalise A431 cells, forming green fluorescent granular appearance in A431 cells as compared with NHDF cells, where no significant fluorescent signal was observed.

### Effect of endosomal inhibitors on the entry of peptide NRPDSAQFWLHH into A431 cells

**Figure 4:**
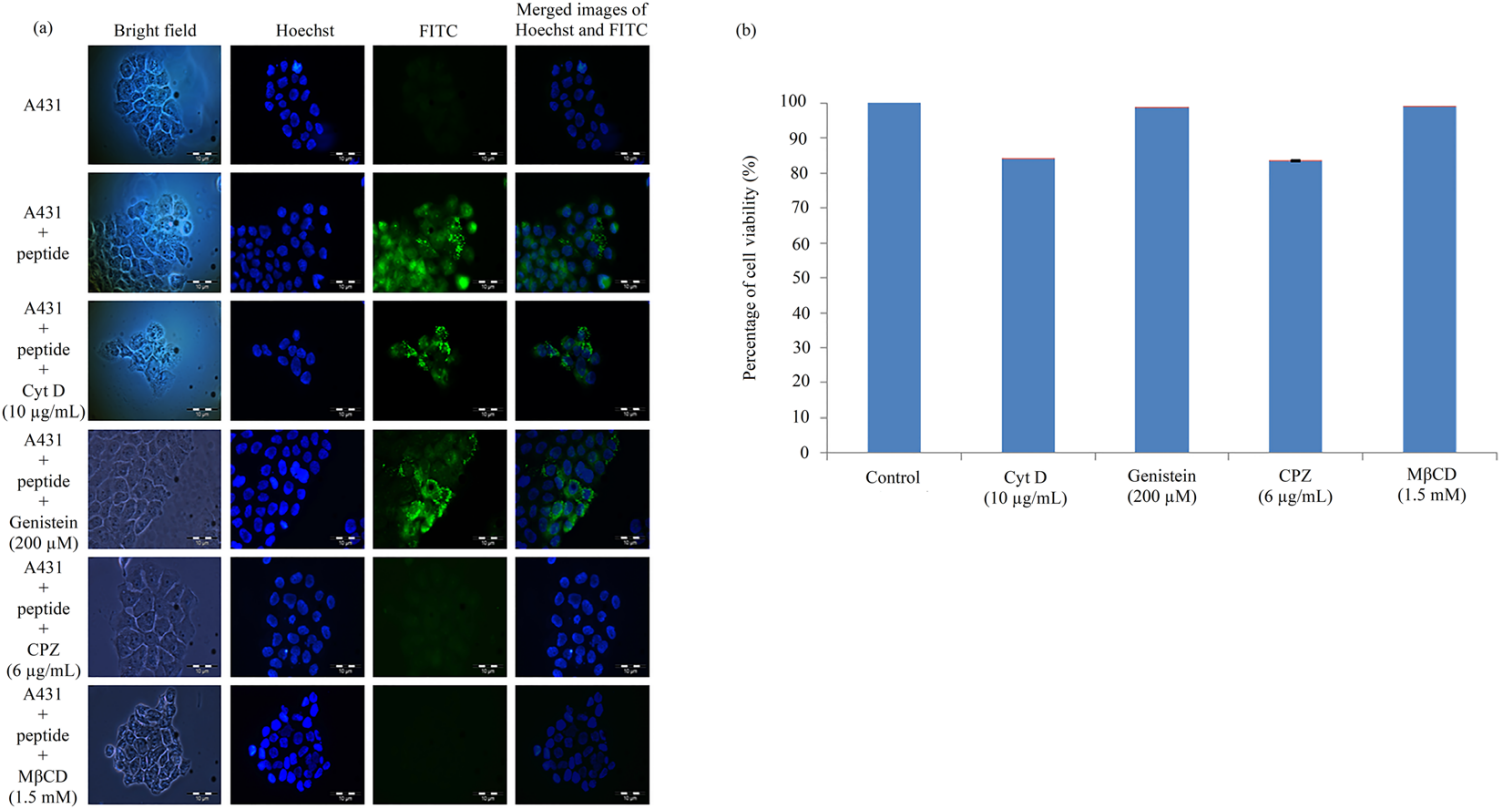
Effect of endosomal inhibitors on the entry of peptide NRPDSAQFWLHH into A431 cells. (**a**) A431 cells were pre-incubated with different endosomal inhibitors, and in the presence of fluorescein-labelled peptide NRPDSAQFWLHH. Endosomal inhibitors are labelled on the left of figure. (**b**) MTT assay showing the viability of A431 cells in the presence of inhibitors. Data are expressed as mean ± standard deviation (n = 3).

Four endocytosis inhibitors which prevent endocytic pathway: methyl-β-cyclodextrin (MβCD), genistein, chlorpromazine hydrochloride (CPZ) and cytochalasin D (Cyt D) were used to study the uptake mechanism of peptide NRPDSAQFWLHH into A431 cells. The cells were pre-incubated with the inhibitors, followed by the addition of peptide NRPDSAQFWLHH, and incubated at 37 °C for 16 h. After the incubation, the cells were observed under a fluorescence microscope. As shown in Fig. 4a, the amount of green granular appearance in A431 cells reduced drastically in the presence of CPZ (6 µg/mL) and MβCD (1.5 mM), suggesting these two inhibitors prevented the internalisation of the peptide into A431 cells. In contrast to CPZ and MβCD, the presence of Cyt D (10 µg/mL) and genistein (200 µM) did not affect the formation of green granules in the cells, indicating Cyt D and genistein did not inhibit internalisation of the peptide into the cells. MTT assay was performed to assess A431 cell’s viability in the presence of Cyt D (10 µg/mL), genistein (200 µM), CPZ (6 µg/mL) and MßCD (1.5 mM). All the treated cells have viability over 83% (Fig. 4b), indicating the treatments with these inhibitors were not toxic to the cells.

### Peptide NRPDSAQFWLHH internalises A431 cells via epithelial growth factor receptor (EGFr)

**Figure 5:**
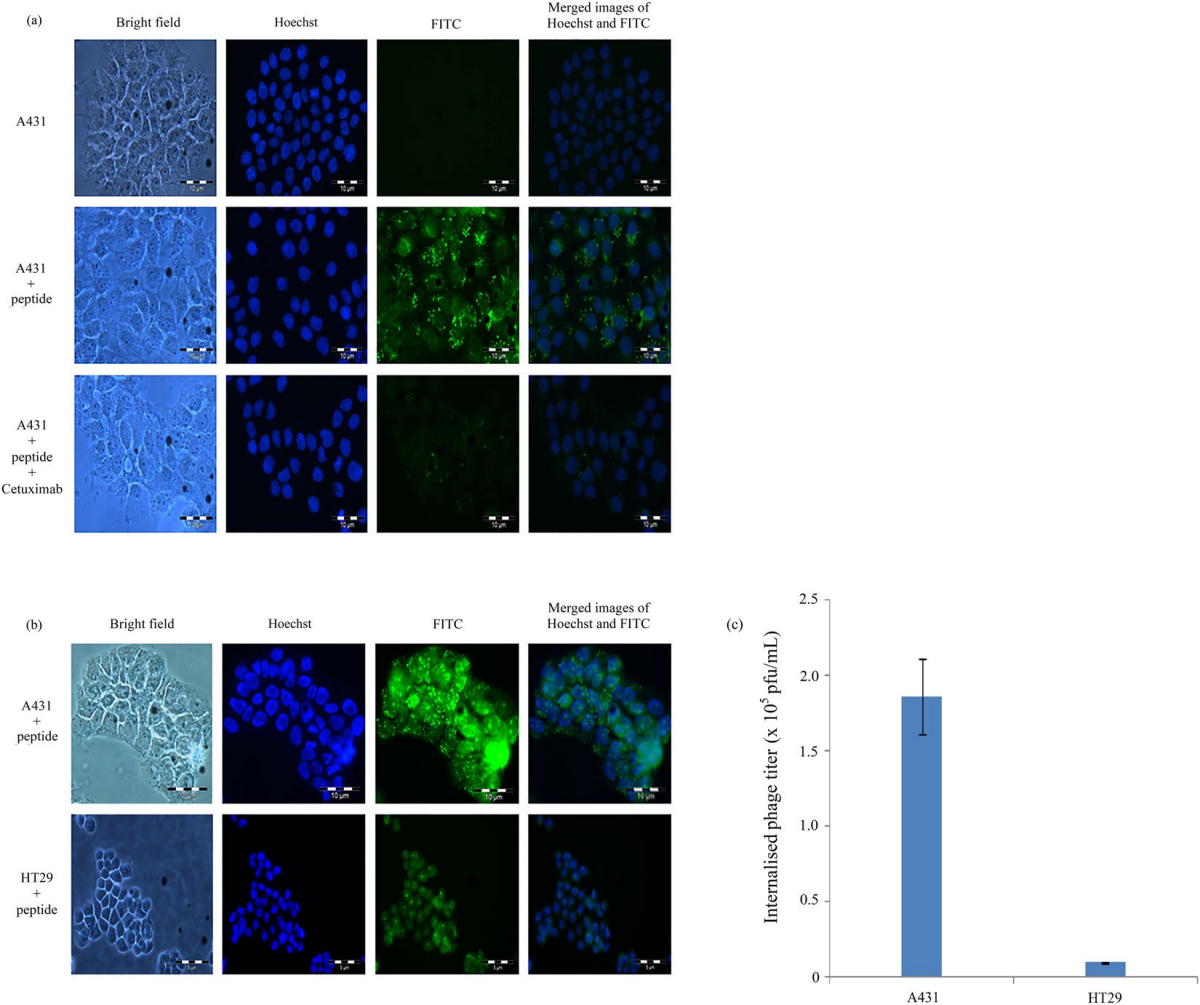
Internalisation of peptide NRPDSAQFWLHH via epithelial growth factor receptor (EGFr). (**a**) Cetuximab inhibits internalisation of peptide into A431 cells. Fluorescein-labelled peptide NRPDSAQFWLHH was added into A431 cells and incubated at 37 °C for 16 h in the presence and absence of anti-EGFr (Cetuximab 225; 10 µg/mL). (**b**) Internalisation of peptide NRPDSAQFWLHH into cells expressing different level of EGFr. Fluorescein-labelled peptide NRPDSAQFWLHH (0.25 mM) was added into A431 and HT29 cells and incubated at 37 °C for 16 h. The peptide internalised A431 cells more than HT29 cells. (**c**) The amount of phage internalised into A431 and HT29 cells after 12 h of incubation at 37 °C, was measured with the phage titration assay as described in the Materials and Methods. Data are expressed as mean ± standard deviation (n = 3).

To determine whether EGFr is responsible for the internalisation of peptide NRPDSAQFWLHH into A431 cells, the cells were co-incubated with the peptide and anti-EGFr antibody, Cetuximab, for 16 h. Figure 5a shows that in the presence of Cetuximab, the internalisation of the peptide into A431 cells was greatly reduced, suggesting the cellular internalisation of the peptide is via EGFr. To further confirm the internalisation of the peptide is via EGFr, A431 (1.2 × 10^6^ EGFr/cell) and HT29 (9 × 10^3^ EGFr/cell) cells expressing different levels of EGFr were incubated with the FITC-labelled peptide. As shown in Fig. 5b, peptide FITC-NRPDSAQFWLHH was taken up efficiently by A431 cells but not HT 29 cells. Phage titration assay showed that the titer of internalised phage in A431 cells was significantly higher (1.86 × 10^5^ pfu/mL) than the phage internalised into HT29 cells (9.50 × 10^3^ pfu/mL; Fig. 5c).

### Conjugation of peptide onto tHBcAg VLNPs and delivery of the nanoparticles into A431 cells

**Figure 6:**
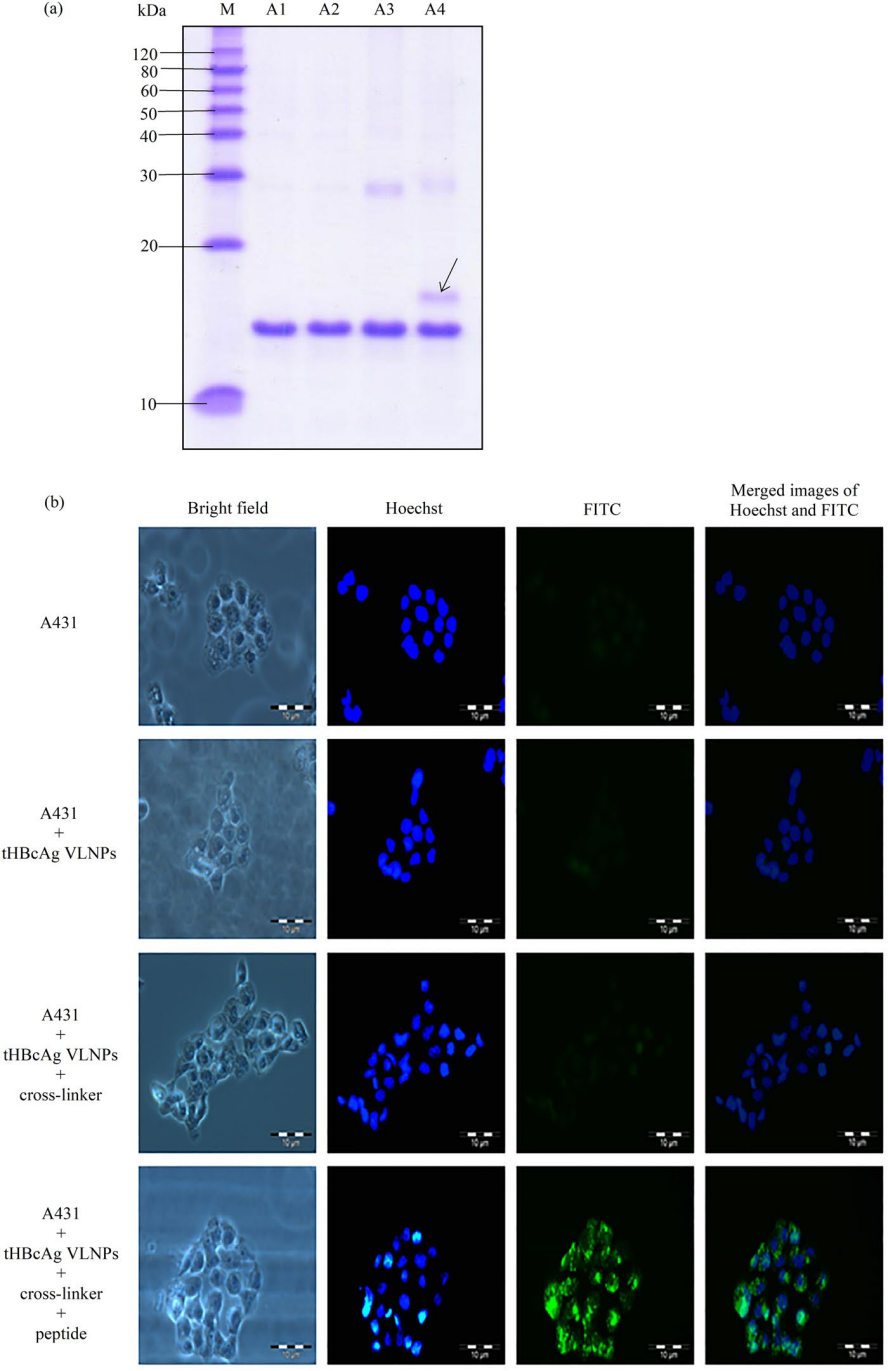
Delivery of tHBcAg VLNPs into A431 cells with peptide NRPDSAQFWLHHGGGSLLGRMKGA containing the nanoglue. (**a**) SDS-PAGE of tHBcAg conjugated to peptide NRPDSAQFWLHHGGGSLLGR-MKGA. Lanes M: molecular mass markers (kDa), A1: tHBcAg, A2: tHBcAg plus peptide NRPDSAQFWLH-HGGGSLLGRMKGA without cross-linker, A3: tHBcAg plus cross-linker without peptide, A4: tHBcAg plus cross-linker and peptide NRPDSAQFWLHHGGGSLLGRMKGA. The arrow shows the tHBcAg monomer conjugated to the peptide. The full length gel is shown in Supplementary Figure S1. (**b**) Delivery of tHBcAg VLNPs conjugated to peptide NRPDSAQFWLHHGGGSLLGRMKGA into A431 cells. tHBcAg was detected with the mouse anti-HBcAg monoclonal antibody, followed by the FITC-conjugated goat anti-mouse antibody. A431 cells incubated with tHBcAg VLNPs that had been conjugated to the peptide showed strong green fluorescent dots. The samples added to the cells are labelled on the left of figure.

The A431 CPP with the sequence NRPDSAQFWLHH was cosynthesised with the nanoglue (SLLGRMKGA) at its C-terminal end, and these sequences were separated by a linker (GGG). The resulting 24-residue peptide, NRPDSAQFWLHHGGGSLLGRMKGA, was conjugated at the spikes of the tHBcAg VLNPs. Figure 6a shows that the tHBcAg monomer cross-linked with the peptide migrated slower than the tHBcAg monomer (17 kDa) in a SDS-polyacrylamide gel, demonstrating the monomer was successfully conjugated with the peptide (the original full-length image of SDS-polyacrylamide gel is shown in Supplementary Figure S1). The internalisation of tHBcAg VLNPs conjugated with the peptide into A431 cells was examined by immunofluorescence microscopy. The tHBcAg was detected by the mouse anti-HBcAg monoclonal antibody and subsequently followed by the FITC conjugated goat anti-mouse antibody (Fig. 6b). This demonstrates that the CPP was biologically active and capable to deliver tHBcAg VLNPs into A431 cells by emitting strong green fluorescent signals.

## Discussion

For the past decade, CPPs have opened new avenues in the field of drug delivery owing to their ability to cross cell membranes and enter into cells. Thus, a phage displayed peptide library with diverse sequences and simple selection procedures has been exploited extensively to isolate CPPs for targeting cells^19^. The CPPs reported in literature internalised hepatocarcinoma^4,20–22^, glioma^14,23^, non-small cell lung carcinoma^24,25^, ovarian tumour^26^, cervical cancer^7^, gastric cancer^9^, oral squamous cell carcinoma (OSCC)^10^, melanoma^11^, and colorectal cancer^12^. In the present study, we successfully isolated a phage clone carrying the peptide sequence NRPDSAQFWLHH which internalised into A431 SCC cells, via subtraction biopanning using a 12-residue phage displayed peptide library. Immunofluorescence microscopy demonstrated internalisation of the selected phage clone into SCC cells where green fluorescent granules were observed in A431 cells (cancerous cell), but not in NHDF cells (normal cell). In addition, the ability of the synthetic peptide NRPDSAQFWLHH to compete with the selected phage clone for the internalisation into A431 cells suggests that the peptide is responsible for internalisation of the selected phage clone without the involvement of the phage coat proteins. As the phage particle is much larger than the synthetic peptide, thus the phage takes a longer time to interact with a receptor, whereby it needs to orientate itself in order for the CPP displayed on the g3p protein to fit into the receptor site. Therefore, in the presence of the synthetic peptide and phage particle, the former reduced the intake of the latter by the A431 cells in a competitive manner. Detection of peptide NRPDSAQFWLHH labelled with fluorescein in A431 cells but not NHDF cells further confirmed internalisation of the peptide into A431 cells.

A thorough understanding of the distinct endocytotic mechanism utilised by a CPP to internalise a target cell is important to determine the intracellular pharmacokinetics and the fate of the CPP. This is essential for optimal design of delivery vehicles for effective delivery of therapeutic molecules to the specific site of action. Active and energy-dependent endocytosis process relies on several proteins and enzymes which are sensitive to temperature, thus this process is reduced by lower temperatures^27^. A drastic reduction in the titer of internalised phage incubated at 4 °C suggests that the internalisation of the isolated phage clone carrying the peptide sequence NRPDSAQFWLHH is an active and energy dependent endocytosis process. However, there are four types of endocytosis: caveolae-mediated endocytosis, clathrin-mediated endocytosis, phagocytosis and macropinocytosis. To understand better the peptide’s internalisation mechanism, four endocytosis inhibitors: Cyt D, genistein, CPZ and MβCD were applied to elucidate the endocytotic pathway of the peptide. Cyt D which interferes with actin polymerisation^28^, showed no noticeable inhibition in the entry of the peptide into A431 cells, indicating that macropinocytosis, an actin-dependent endocytotic mechanism^29^, was not favoured for the peptide internalisation. Genistein inhibits caveolae-mediated endocytosis by disrupting actin network and recruitment of dynamin II at the site of endocytosis^30,31^. In this study, it is clearly demonstrated that the internalisation of peptide NRPDSAQFWLHH into A431 cells is not inhibited by genistein, suggesting that the peptide internalisation is not caveolae dependent. MβCD is commonly used to inhibit the formation of clathrin coated endocytotic vesicles^32,33^. In this study, internalisation of peptide NRPDSAQFWLHH was almost completely abolished by MβCD, suggesting that clathrin mediated endocytosis plays a vital role in the peptide internalisation. To verify the involvement of clathrin-mediated endocytosis, we evaluated the effect of CPZ on the peptide internalisation. CPZ inhibits clathrin-coated pit formation^34^, and in the present study, treatment of A431 cells with CPZ inhibited the formation of green fluorescence in the cells. This verifies that internalisation of peptide NRPDSAQFWLHH is via clathrin mediated endocytosis.

Cancer cells often overexpress tumour associated antigens (TAA) or tumour specific antigens (TSA). These antigens represent potential sites for delivery of therapeutic agents^4^. Short ligands or peptides which can bind specifically to the active or biological relevant sites on target proteins can enhance the efficacy of tumour targeting therapy^16,17^. Studies on anti-cancer CPPs mostly focus on integrins due to their important roles in the initiation, progression and metastasis of tumours, which include the head and neck squamous cell carcinoma^10^, brain tumour^35,36^, hepatocarcinoma^37^, prostate cancer^38^, colon cancer^39^, and brain metastasis^40^. However, studies on CPPs targeting skin cells were mainly focused on the transdermal delivery and uptake of enzyme, si-RNAs, plasmid DNA and nanoparticles in normal skin cells^41–51^. In the present study, we successfully isolated a novel CPP which internalised A431 SCC cells, and this peptide can be used as a ligand to target nanoparticles to the skin cancer cells.

EGFr regulates normal growth and differentiation of cells but dysregulation of EGFr or its ligands play a role in tumorigenesis of cells^52^. Many tumours that originate from epithelial tissues, including SCC, non-small cell lung cancer, gastric, esophageal, colorectal, prostate, renal, bladder, ovarian, and pancreatic cancers are found to be associated with EGFr overexpression^53,54^. The ligand-induced EGFr endocytosis is believed to be mediated by the clathrin^54^. In this study, we demonstrated that peptide NRPDSAQFWLHH internalised A431 cells via clathrin. Since EGFr is overexpressed in A431 cells^55–59^ (approximately 1.2 × 10^6^ EGFr/cell)^53^, we speculated that peptide NRPDSAQFWLHH could internalise into A431 cells through the EGFr. An anti-EGFr antibody, Cetuximab C225, was used to evaluate the involvement of EGFr in the peptide internalisation. Depletion of green fluorescent granules in A431 cells co-incubated with FITC-NRPDSAQFWLHH and Cetuximab C225 further demonstrated the involvement of EGFr in the peptide internalisation. In addition, the specificity of peptide FITC-NRPDSAQFWLHH was tested on another cancer cell line, HT-29, with a lower EGFr expression level (9 × 10^3^ EGFr/cell)^53^, and no significant green fluorescent granules were observed. Phage titration assay showed a significant reduction of the internalised phage titer in HT29 compared to A431 cells. Collectively, the results strongly suggest that peptide NRPDSAQFWLHH internalised into A431 cells through EGFr.

tHBcAg VLNPs have been employed as vehicles for the delivery of GFP, oligonucleotides, and DOX into HepG2^15^, HeLa^7^ and colorectal cancer cells^16^, respectively. These studies employed the nanoglue to display the HeLa-cell CPP^7^ and folic acid molecules^16^ on the surface of tHBcAg VLNPs. To demonstrate the potential of peptide NRPDSAQFWLHH as a ligand to deliver VLNPs into A431 cells, this peptide was synthesised at the N-terminal end of the nanoglue and conjugated to tHBcAg VLNPs (Supplementary Figure S2). Fluorescence microscopy revealed that the tHBcAg VLNPs displaying peptide NRPDSAQFWLHH via the nanoglue successfully delivered the VLNPs into A431 cells.

The isolated CPP can be used in topical application, in which the peptide can serve as a targeting ligand by conjugating with nanoparticles. Loading of drugs into the CPP-conjugated nanoparticles would increase the efficacy of the drugs, and at the same time reduce their toxicity. In addition, the CPP can be used in systemic delivery, but the peptide has to be modified to improve its *in vivo* stability. This could be achieved by constraining the primary structure of the peptide into a cyclic form^60^, and replacing the amino acid residues with D-amino acids or their analogues which are resistant to endogenous protease activities^61–63^.

In summary, a novel CPP with the sequence NRPDSAQFWLHH, internalising A431 SCC cells via clathrin mediated endocytosis and EGFr was isolated in this study. Apart from SCC, this peptide ligand of EGFr has potential applications in targeting treatments of patients with EGFr-positive malignancies, which include non-small cell lung cancer, esophageal, gastric, prostate, colorectal, bladder, pancreatic, ovarian and renal cancers. We also demonstrated that the peptide can be used to target and deliver tHBcAg VLNPs into A431 cells. This paves the way for delivering drugs, nucleic acids and molecules into cells overexpressing EGFr. The application of this peptide is not limited as a ligand to target and internalise VLNPs into cells, it can also be incorporated into liposomes and other nanoparticles for a broader application in nanomedicine and targeting cancer imaging.

## Materials and Methods

### Cell culture

Human squamous carcinoma cell line (A431) and human colorectal cell line (HT29) were obtained from the American Type Culture Collection (ATCC), while normal human dermal fibroblast cell line (NHDF) was obtained from LONZA (Tuas, Singapore). A431 and HT29 cells were cultured in Dulbecco’s Modified Eagle’s medium (DMEM) (Sigma Aldrich, St. Louis, Missouri, USA) containing 10% (v/v) fetal bovine serum (FBS). NHDF cells were cultured in fibroblast basal medium (FBM) containing 2% (v/v) FBS, 1% (v/v) insulin, 1% (v/v) hFGF-β and 1% (v/v) gentamicin/amphotericin-B. All cells were cultured at 37 °C in a humidified atmosphere containing 5% CO_2_.

### Subtraction biopanning for selection of A431 cell penetrating peptides (CPPs)

A431 CPPs were isolated by subtraction biopanning using the 12-residue phage displayed peptide library (New England BioLabs Inc., Ipswich, Massachusetts, USA) against NHDF followed by biopanning against A431 cells. NHDF cells (1 × 10^6^ cells) and A431 cells (1 × 10^6^ cells) were seeded separately onto a 10 cm-tissue culture dish in FBM (5 mL) and DMEM (5 mL), respectively. The cells were incubated overnight at 37 °C in a humidified CO_2_ incubator until they reached subconfluent monolayer. After changing the medium, the NHDF cells were added with phages (1 × 10^11^ pfu) and incubated at 37 °C for 12 h. The phage suspension subtracted from NHDF cells was then centrifuged for 10 min at 500 × g to precipitate the cells. The supernatant was added to A431 cells and incubated at 37 °C for another 12 h. The infected A431 cells were washed 6 times with phosphate buffered saline (PBS pH 7.4: 2.7 mM KCl, 137 mM NaCl, 8.1 mM Na_2_HPO_4_, 1.47 mM KH_2_PO_4_) after removing the medium. The cells were trypsinised and resuspended in PBS containing proteinase K (0.1 mg/mL) prior to incubation for 1 h at 4 °C. The cells were then washed with ice cold PBS (10 mL) twice and resuspended in TBS buffer pH 8.0 (500 µL: 150 mM NaCl, 50 mM Tris-HCl). After that, lysis buffer pH 8.0 [500 µL: 10 mM Tris-HCl, 2 mM ethylenediaminetetraacetic acid (EDTA), and 2% (w/v) sodium deoxycholate] was added and the cells were lysed by vortexing. The phages in the cell lysate were propagated in *E*. *coli* strain ER2738 and used in subsequent subtraction biopanning for another two rounds as described above. The titer of infectious phages was determined via phage titration assay by plating out an aliquot of the cell lysate recovered from each round of biopanning using *E*. *coli* strain ER2738 as a host. After 3 rounds of panning, phage DNA was extracted and the nucleotide sequence was determined as described in Tan *et al*.^64^.

### Analysis of the internalisation of the selected M13 phage into A431 cells with immunofluorescence microscopy

The A431-internalising phage selected from biopanning was tested on A431 and NHDF cells in order to evaluate the specificity of the phage towards these cells. NHDF and A431 cells (200,000 cells/well) were seeded separately onto sterile glass cover slips in a six-well plate and incubated overnight at 37 °C. After changing the medium, the cells were added with the purified phage (1 × 10^12^ pfu) and incubated at 37 °C for another 72 h. The cells were then washed 5 times with PBS and fixed with paraformaldehyde [3.7% (w/v)] for 10 min. After fixation, the cells were permeabilised using ice cold methanol at −20 °C for 6 min prior to incubation with the mouse anti-M13 monoclonal antibody [1:100 dilutions in PBS supplemented with bovine serum albumin (BSA; 0.2 mg/mL); GE Healthcare Life Sciences, Little Chalfont, Buckinghamshire, U.K.] at room temperature (RT) for 1 h. The goat anti-mouse IgG conjugated to Alexa-Fluor^®^ 488 [1: 100 dilutions in PBS supplemented with BSA (0.2 mg/mL); Abcam, Cambridge, Massachusetts, USA] was then added and incubated for another 1 h at RT. Hoechst 33342 (Ex_360nm_ and Em_460nm_; 2 drops/mL in PBS; Life Technologies, Carlsbad, California, USA) was used to stain the cell nuclei for 10 min. After that, the cells were washed thoroughly with PBS. A drop of mounting medium [0.1 M propyl gallate, 90% (v/v) glycerol, 20 mM Tris-HCl (pH 8.5)] was then added onto a glass slide, the cover slip was mounted and sealed with nail polish. The cells were examined under an Olympus X5 fluorescence microscope.

### Analysis of energy-dependent uptake of the selected M13 phage

A431 cells (200,000 cells/well) were seeded onto sterile glass coverslips which had been placed in a six-well plate, and incubated overnight at 37 °C. Energy-dependent uptake experiments were performed by pre-incubating the cells for 30 min at 4 °C prior to the addition of the selected phage. After the pre-incubation, the purified phage (1 × 10^12^ pfu) was added to the cells, and incubated in parallel at 37 °C and 4 °C for 6 h. The cells were then washed, fixed and permeabilised with ice cold methanol prior to incubation with the mouse anti-M13 monoclonal antibody followed with the goat anti-mouse IgG conjugated to Alexa-Fluor^®^ 488 as described above. Hoechst 33342 was used to counterstain the cell nuclei. The cells were then washed, mounted onto a glass slide, sealed with nail polish and examined with a fluorescence microscope. The phage internalised into A431 cells (1 × 10^6^ cells/mL) at 37 °C and 4 °C was measured with phage titration assay by plating out an aliquot of the cell lysate using *E*. *coli* strain ER2738 as a host.

### Peptide-phage competitive assay

A431 cells (1 × 10^6^ cells) were seeded onto a 10 cm-tissue culture dish containing DMEM (5 mL). After incubation, the medium was changed. The purified phage (1 × 10^12^ pfu) together with the corresponding synthetic peptide, NRPDSAQFWLHH (0.25 mM; Mimotopes Pty Ltd, Clayton, Victoria, Australia) were added to the A431 cells and incubated for 12 h at 37 °C. The titer of the internalised phage was determined by phage titration assay as described above. In this experiment, the cells added with the selected phage and unrelated peptides (MHRSLLGRMKGA, VSRHQSWHPHDL and HTKQIPRHIYSA) served as negative controls.

### Selective internalisation property of peptide NRPDSAQFWLHH

The selective internalisation property of peptide NRPDSAQFWLHH was tested on NHDF and A431 cells. These cells (200,000 cells/well) were seeded separately onto sterile glass coverslips which had been placed in a six-well plate, and incubated overnight at 37 °C. After changing the medium, the cells were added with FITC-conjugated peptide (FITC-NRPDSAQFWLHH; 0.25 mM; Mimotopes Pty Ltd, Clayton, Victoria, Australia), and incubated for 16 h at 37 °C. The cells were then washed 3 times with PBS and fixed with paraformaldehyde [3.7% (w/v)]. Hoechst 33342 was added and incubated for 10 min to stain the cell nuclei. The cells were mounted onto a glass slide and observed under a fluorescence microscope.

### Endocytosis inhibitory assay

Four endocytosis inhibitors [Cyt D (10 µg/mL; Calbiochem, San Diego, California, USA), CPZ (6 µg/mL; Santa Cruz Biotechnology, Dallas, Texas, USA), MβCD (1.5 mM; Sigma Aldrich, St. Louis, Missouri, USA) and genistein (200 µM; Calbiochem, San Diego, California, USA)] were used to study the energy-dependent endocytosis of peptide NRPDSAQFWLHH into A431 cells. The cells (200,000 cells/well) were seeded onto sterile glass coverslips which had been placed in a six-well plate, and incubated at 37 °C for overnight. The medium was then removed and the cells were pretreated with the inhibitors at 37 °C for 30 min. After the pretreatment with inhibitors, FITC-NRPDSAQFWLHH (0.25 mM) was added to the cells and incubated for 16 h at 37 °C in the presence of inhibitors. After removing the medium, the cells were washed, fixed and stained with Hoechst 33342. The cells were mounted onto a glass slide and examined under a fluorescence microscope.

### Inhibition of peptide internalisation into A431 cells using anti-epidermal growth factor receptor (anti-EGFr) Cetuximab

A431 cells (200,000 cells/well) were seeded onto sterile glass coverslips in a six-well plate and incubated overnight at 37 °C. The medium was then removed and the cells were pre-incubated with 10 µg/mL anti-EGFR (Cetuximab clone C225; Merck, Billerica, Massachusetts, USA) for 30 min. After the pre-incubation, FITC-NRPDSAQFWLHH (0.25 mM) was added to the cells and incubated for 16 h at 37 °C in the presence of anti-EGFR (10 µg/mL). After removing the medium, the cells were washed, fixed, stained with Hoechst 33342, and viewed under a fluorescence microscope.

### Incubation of FITC-NRPDSAQFWLHH peptide in cell lines expressing different levels of EGFr

A431 and HT29 cell lines (200,000 cells/well) were seeded onto sterile glass coverslips in a six-well plate and incubated overnight at 37 °C. After changing the medium, peptide FITC-NRPDSAQFWLHH (0.25 mM) was added to the cell lines and incubated at 37 °C for 16 h in a CO_2_ incubator. The cells were then washed, fixed, stained with Hoechst 33342, and viewed under a fluorescence microscope. The amount of phage internalised into A431 and HT29 cells (1 × 10^6^ cells) which had been incubated with the purified phage (1 × 10^12^ pfu) at 37 °C for 12 h was measured with the phage titration assay as described above.

### Conjugation of peptide NRPDSAQFWLHHGGGSLLGRMKGA to tHBcAg nanoparticles

Production and purification of tHBcAg are as described in Tan *et al*.^65^ and Yoon *et al*.^66^. Conjugation of peptide NRPDSAQFWLHHGGGSLLGRMKGA (Mimotopes Pty Ltd, Clayton, Victoria, Australia) to tHBcAg nanoparticles was done by incubating tHBcAg: peptide (1:1 ratio) in phosphate buffer containing conjugation reagents as described in Lee *et al*.^7^ and Tang *et al*.^18^, with some modifications. The peptide and tHBcAg (60 µg) were incubated at 4 °C for 8 h in 120 µL of reaction buffer containing sulfo-NHS (1.8 mM), NaH_2_PO_4_/Na_2_HPO_4_ (pH 7; 25 mM), and EDC (1.8 mM). The conjugation of peptide to tHBcAg nanoparticles was analysed with SDS-PAGE.

### Delivery of tHBcAg nanoparticles conjugated with peptide NRPDSAQFWLHHGGGSLLGRMKGA into A431 cells

To evaluate the internalisation of the tHBcAg nanoparticles conjugated with peptide NRPDSAQFWLHHGGGSLLGRMKGA into A431 cells, the cross-linking sample was dialysed against phosphate buffer (pH 7), concentrated with VIVASPIN 6 (30 kDa cut-off polyethersulfone membrane; VIVASCIENCE, Germany) at 4500 × *g*, 4 °C and added to A431 cells (250 µg). After incubation at 37 °C for 16 h, the cells were washed, fixed and permeabilised prior to incubation with the mouse anti-HBcAg monoclonal antibody [1:100 dilutions in PBS containing BSA (0.2 mg/mL); Santa Cruz Biotechnology, Dallas, Texas, USA] for 1 h at RT. Then, the FITC-conjugated goat anti-mouse antibody (1:100 dilutions in PBS containing 0.2 mg/mL BSA; BD Biosciences, San Jose, California, USA) was added and incubated for another 1 h at RT. The cells were then washed, stained with Hoechst 33342 and viewed under a fluorescence microscope.

### Statistical Analysis

The SPSS programme was used in statistical analysis. Values of p < 0.01 are considered to be significant.

## Electronic supplementary material

Supplementary Information
