# Supplementary material for: Targeted Delivery of Cell Penetrating Peptide Virus-like Nanoparticles to Skin Cancer Cells
Source: Sci Rep. 2018 May 31;8:8499. doi: 10.1038/s41598-018-26749-y (PMC5981617; doi:10.1038/s41598-018-26749-y)
Supplement: Supplementary file 1 — Supplementary Information [file 41598_2018_26749_MOESM1_ESM.pdf]

Targeted Delivery of Cell Penetrating Peptide Virus-like Nanoparticles to Skin Cancer Cells

Bee Koon Gan[1], Chean Yeah Yong[1], Kok Lian Ho[2], Abdul Rahman Omar[1,3], Noorjahan Banu Alitheen[1,4] & Wen Siang Tan[1,5*]

[1]Institute of Bioscience, Universiti Putra Malaysia, 43400 UPM Serdang, Selangor, Malaysia.

[2]Department of Pathology, Faculty of Medicine and Health Sciences, Universiti Putra Malaysia, 43400 UPM Serdang, Selangor, Malaysia.

[3]Department of Veterinary Pathology & Microbiology, Faculty of Veterinary Medicine, Universiti Putra Malaysia, 43400 UPM Serdang, Selangor, Malaysia.

[4]Department of Cell and Molecular Biology, Faculty of Biotechnology and Biomolecular Sciences, Universiti Putra Malaysia, 43400 UPM, Serdang Selangor, Malaysia.

[5]Department of Microbiology, Faculty of Biotechnology and Biomolecular Sciences, Universiti Putra Malaysia, 43400 UPM, Serdang Selangor, Malaysia.

*Correspondence and requests for materials should be addressed to W.S.T. (email: wstan@upm.edu.my)

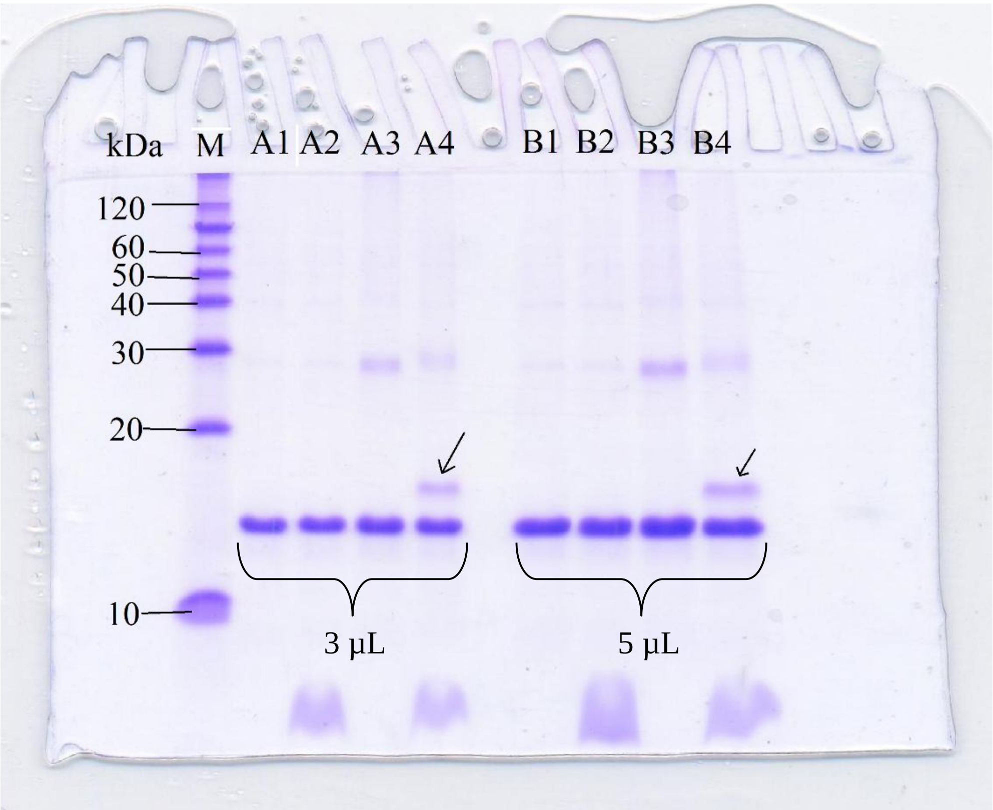

Figure S1. Full length SDS-polyacrylamide gel of tHBcAg conjugated to peptide NRPDSAQFWLHHGGGSLLGRMKGA. The tHBcAg conjugated to peptide NRPDSAQFWLHHGGGSLLGRMKGA was electrophoresed on 15% (w/v) SDS-polyacrylamide gel, and stained with CBB-R250. **Lanes M:** molecular mass markers (kDa), **A1 & B1:** tHBcAg, **A2 & B2:** tHBcAg plus peptide NRPDSAQFWLHHGGG-SLLGRMKGA without cross-linker, **A3 & B3:** tHBcAg plus cross-linker without peptide, **A4 & B4**: tHBcAg plus cross-linker and peptide NRPDSAQFWLHHGGGSLLGRMKGA. The sample volume loaded in lanes **A1-A4** was 3 µL; while the sample volume loaded in lanes **B1-B4** was 5 µL. The arrows show the tHBcAg monomer conjugated to the peptide.

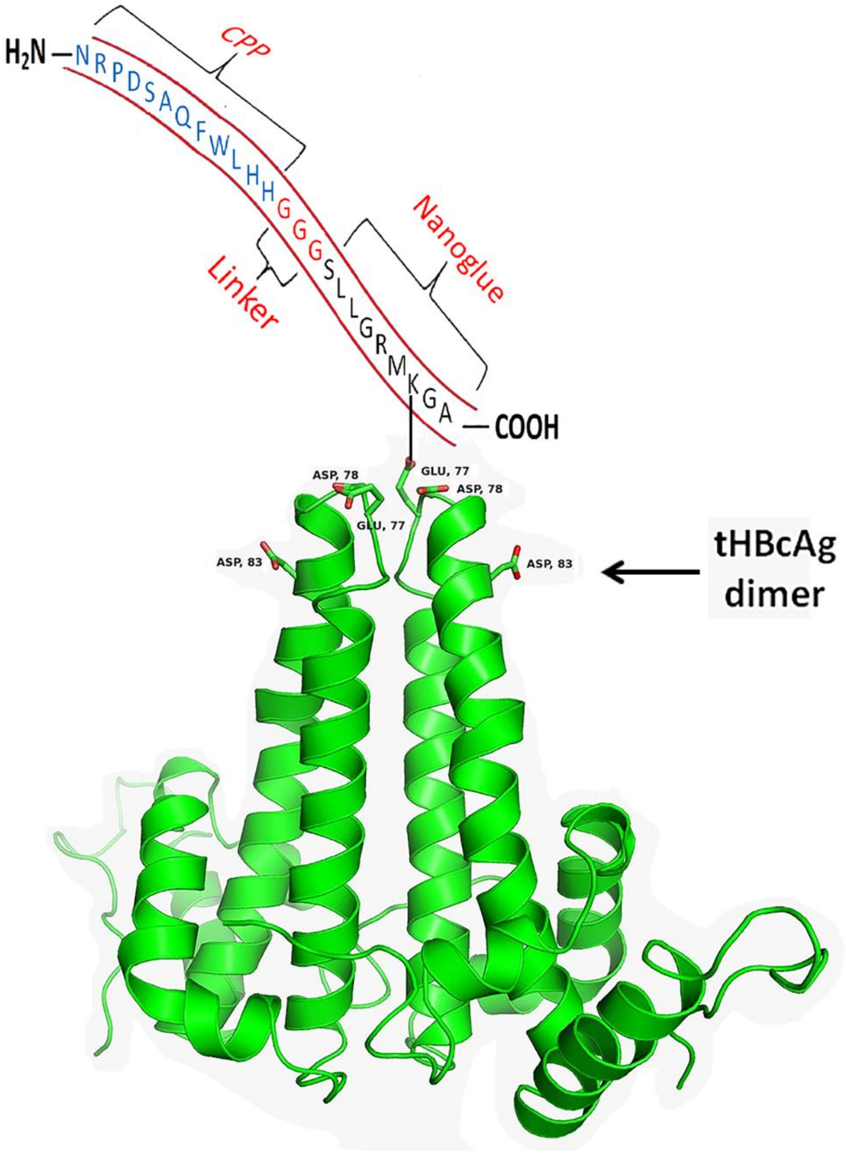

Figure S2. Conjugation of peptide NRPDSAQFWLHHGGGSLLGRMKGA at the tip of tHBcAg dimer. The 24-residue peptide contains the CPP (NRPDSAQFWLHH), a linker (GGG), and the nanoglue (SLLGRMKGA). The nanoglue binds specifically at the tip of tHBcAg dimer, and the primary amine group from lysine residue of the nanoglue was cross-linked to the adjacent carboxyl group from either glutamic acid (77) or aspartic acid (78) residues located at the tip of tHBcAg dimer, in the presence of the zero-length cross-linker EDC and sulfo-NHS.
